# Association between Blood Cadmium Levels and 10-Year Coronary Heart Disease Risk in the General Korean Population: The Korean National Health and Nutrition Examination Survey 2008–2010

**DOI:** 10.1371/journal.pone.0111909

**Published:** 2014-11-10

**Authors:** Jun-Pyo Myong, Hyoung-Ryoul Kim, Tae-Won Jang, Hye Eun Lee, Jung-Wan Koo

**Affiliations:** 1 Department of Occupational & Environmental Medicine, Seoul St. Mary's Hospital, College of Medicine, The Catholic University of Korea, Seoul, Republic of Korea; 2 Center for Occupational and Environmental Medicine, Seoul St. Mary's Hospital, The Catholic University of Korea, Seoul, Republic of Korea; Stony Brook University, Graduate Program in Public Health, United States of America

## Abstract

**Background:**

Non-occupational heavy metals are considered risk factors for coronary heart disease (CHD). Several recent epidemiologic studies have evaluated the relationship between non-occupational cadmium exposure and risk factors for cardiovascular disease (CVD). This study was designed to investigate the relationship between non-occupational cadmium exposure and risk factors for CHD using the Framingham estimate of 10 year CHD risk.

**Methods:**

The heavy metal dataset of the Korean National Health and Nutrition Examination Survey for 2008 through 2010, a cross-sectional survey of a representative sample of 4,668 non-institutionalized Koreans, was analyzed. Subjects were stratified into seven age groups to minimize the effects of age. The log-transformed blood cadmium concentrations were compared with the Framingham estimate of 10 year CHD risk in each age stratum.

**Results:**

The Framingham estimate of 10 year CHD risk was significantly associated with the log-transformed blood cadmium concentrations (*p*<0.05) in all age groups of Korean men, with the lowest regression coefficient (0.254) for men aged 20 to <35 years and the highest (3.354) for men aged 55 to <60 years; similar results, however, were not observed in Korean women. After adjusting for survey year, age, and urinary cotinine concentration, the log-transformed blood cadmium levels among men aged 20 to <35, 40 to <45, 50 to <55, and 60 to <65 years were significantly associated with systolic blood pressure (*p*<0.05), but not with total and high density lipoprotein (HDL) cholesterol concentrations.

**Conclusions:**

Cadmium exposure, even at non-occupational levels, may be associated with CHD risk in men. Despite the declines in non-occupational cadmium exposure over the past several decades, more efforts are needed.

## Introduction

Cardiovascular disease (CVD), including coronary heart disease (CHD), cardiomyopathy, heart failure, hypertension, and valvular heart disease, is a leading cause of death worldwide, with 17.3 million people worldwide dying of CVD in 2008, including 7.3 million people who died of CHD [1]. CVD is also a leading cause of death in South Korea, where the death rate in 2010 was 112.5 per 100,000, and the death rate for CHD was 47.2 per 100,000 [2].

Smoking, hypertension, hypercholesterolemia, and other factors (age, sex, and air pollution) are among the major risk factors for CHD [3]. In addition, non-occupational exposure to several heavy metals has been associated with CHD [4]. Lead, which is both hepatotoxic and nephrotoxic, may induce cholesterogenesis and phosphogelipidosis in tissue [5], [6]. Blood cadmium level was found to be positively associated with systolic and diastolic blood pressure in the general Korean population [7]. In a US study using data from the National Health and Nutrition Examination Survey (NHANES), blood cadmium was found to positively correlate with peripheral artery disease [8]. Few studies, however, have evaluated the associations between heavy metal concentrations in blood and overall estimates of CHD risk [9], [10].

The Framingham risk score, based on the Framingham Heart Study [11], can be calculated using various factors, including age, sex, systolic blood pressure, total cholesterol and high-density lipoprotein (HDL) cholesterol concentrations, and smoking. Ten year CHD risk categories were classified as >20%, 10–20%, and <10%. However, only one study, in Swedish females aged ≥70 years, has shown an association between blood cadmium concentrations and Framingham risk score (regression coefficient: 0.98, *p*-value  = 0.0001), but even that study could not elucidate a relationship in those aged <70 years [10]. Therefore, it is necessary to evaluate the relationship between the blood cadmium concentrations and CHD risk in persons aged <70 years old. This study was therefore designed to investigate the relationship between blood cadmium concentrations and the Framingham estimate of 10 year CHD risk and its components among the general Korean population aged 25–64 years.

## Materials and Methods

### Data source and study subjects

The heavy metal dataset of the Korean National Health and Nutrition Examination Survey (KNHANES) IV and V (from 2008 through 2010), a representative annual survey of the health, nutritional status, and blood heavy metal concentrations in the civilian, non-institutionalized Korean general population, was analyzed. Representative non-institutionalized Koreans were selected using a stratified multistage clustered probability design developed by the Korea Center for Disease Control and Prevention [12]. Subjects were assessed for blood heavy metal concentrations, within 200 primary sampling units (PSU) in 2008 and 2009, and 192 PSU in 2010. Ten to twelve individuals from each PSU were randomly selected while maintaining a uniform distribution across gender. Five age groups (20–29, 30–39, 40–49, 50–59, and ≥60 years) were assessed in 2008 and 2009, and six age groups (<20, 20–29, 30–39, 40–49, 50–59, and ≥60 years) in 2010. A total of 2,006, 1,991, and 2,355 subjects participated in the KNHANES in 2008, 2009, and 2010, respectively. Participants without laboratory test results (blood chemistry: HDL cholesterol and total cholesterol; urinary cotinine) were excluded (n = 7; 2 in 2008, 3 in 2009, and 2 in 2010), as were those aged ≥65 and ≤25 (n = 1,658; 449 in 2008, 435 in 2009, and 774 in 2010, respectively), leaving a total of 4,688 subjects. The study design was approved by the Institutional Review Board of the Catholic University of Korea, College of Medicine (approval ID: KC12EIS0545).

### Blood Cadmium

Blood cadmium concentrations were measured by the NEODIN Medical Institute, which was certified by the Korean Ministry of Health and Welfare. There was no background cadmium contamination at collection or in storage materials [12]. Cadmium concentrations in whole blood were assessed by graphite furnace atomic absorption spectrometry using a PerkinElmer AAnalyst AAS-600 (PerkinElmer, Turku, Finland), with Zeeman correction. For internal quality assurance and control, commercial standards (Lyphochek Whole Blood Metals, Bio-Rad, CA, USA) were used as reference materials. The coefficients of variation for blood cadmium were 1.51–11.89%, 1.35–5.29%, and 0.97–9.68% in 2008, 2009, and 2010, respectively. For external quality assurance, both the German External Quality Assessment Scheme (G-EQUAS), operated by Friedrich Alexander University, and the quality assurance program, operated by the Korea Occupational Safety and Health Agency (KOSHA), were used in 2008. Quality assurance by the National Institute of Environmental Research (NIER) was added in 2009, and by the Lead and Multielement Proficiency Program (LAMP) of the Centers for Disease Control and Prevention (CDC) in 2010. The limits of detection for blood cadmium in 2008, 2009, and 2010 were 0.056, 0.087, and 0.062 µg/L, respectively. No subject had a blood cadmium concentration below the detection limit.

### Systolic blood pressure, clinical laboratory tests, and hypertension treatment

After 10 minutes of rest, blood pressure was measured three times at 5 minute intervals with a mercury sphygmomanometer while subjects were in a seated position. The first result was discarded, and the next two were used to calculate the average systolic blood pressure. Blood samples for clinical laboratory tests (total cholesterol and HDL cholesterol) were drawn by skilled practitioners after an overnight fast for >10 hours, and total cholesterol and HDL cholesterol were measured using a Hitachi Automatic Analyzer 7600 (Hitachi, Tokyo, Japan). The limits of detection for urinary cotinine and cholesterol were 0.25 µg/L and 33 mg/dL, respectively. Antihypertensive treatment was assessed by answers to the question ‘Are you taking antihypertensive drugs or other treatment?’ Participants who answered ‘yes’ to this question were considered to be taking antihypertensive treatment. Age, systolic blood pressure, and HDL cholesterol levels were categorized by the formula from the Framingham point score [3].

### Urinary cotinine and smoking verification

Spot urinary samples were collected for measurement of urinary cotinine by gas chromatography and mass spectrometry using a PerkinElmer Clarus 600T, with a limit of detection of 1.26 ng/ml. For internal quality assurance and control purposes, standard reference materials were used (ClinChek, RECIPE, Munich, Germany), which showed that the coefficients of variation for urinary cotinine in 2008, 2009, and 2010 were 3.43–9.11%, 0.79–8.17%, and 0.48–5.29%, respectively. The G-EQUAS utilizes a standard protocol to measure urinary cotinine. Individuals with urinary cotinine ≥50 ng/mL were defined as cotinine-verified smokers [13]–[15].

### Framingham estimate of 10-year coronary heart disease (CHD) risk

The Framingham estimate of 10 year risk of CHD was derived from the Framingham point score, based on age, HDL cholesterol and total cholesterol concentrations, systolic blood pressure, and smoking by gender [11]. The Framingham risk score is widely used to predict the 10 year CHD risk of an individual. Total points of risk factors ranged from 0∼17 in males and 1∼25 in females, with both representing Framingham point score ranges of 1% to 30%.

### Statistical analysis

All statistical analyses were performed using SAS 9.2 (SAS institute, Cary, NC, USA). Analyses were designed to account for the stratified multistage clustered probability design and weights in KNHANES (2008 through 2010). The distribution of cadmium in blood was skewed and was therefore log-transformed to estimate geometric mean (GM) and geometric standard error (GSE). To control for confounders on both the Framingham estimate of 10 year CHD risk and blood cadmium levels, age and gender were stratified for all analyses. Cadmium levels were classified into quartiles within each age category. To determine the association between the Framingham estimate of 10 year CHD risk and blood cadmium levels, the log-transformed blood cadmium concentrations were regressed against the Framingham estimate of 10 year CHD risk in each of the seven age groups, 20 to <35, 35 to <40, 40 to <45, 45 to <50, 50 to <55, 55 to <60, and 60 to <65 years, after adjusting for survey years. To evaluate the associations between log-transformed blood cadmium levels and the components of the Framingham points scores (systolic blood pressure, total cholesterol, and HDL cholesterol), multiple survey regression analyses were performed after adjusting for survey years, age, and urinary cotinine levels (SAS Syntax: PROC SURVEYREG).

## Results

The GMs and GSEs of cadmium in blood are presented by sex in Table 1. The GMs of blood cadmium levels were higher in older participants and in those with higher systolic blood pressure, higher total cholesterol, and higher urinary cotinine concentrations.

**Table 1:** Distribution in blood cadmium levels (µg/L) by sex.

				Men			Women	
			N	GM	GSE	N	GM	GSE
Age (years)								
		25–34	595	0.73	1.03	615	0.74	1.03
		35–39	350	0.88	1.03	328	0.98	1.03
		40–44	316	0.93	1.03	286	1.11	1.03
		45–49	282	1.01	1.04	312	1.30	1.03
		50–54	306	1.02	1.04	315	1.37	1.03
		55–59	285	1.13	1.04	285	1.39	1.03
		60–64	217	1.11	1.04	196	1.30	1.04
Systolic Blood Pressure (mmHg)								
		<120	1218	0.84	1.02	1619	0.98	1.02
		120–129	545	0.92	1.03	339	1.19	1.03
		130–139	335	1.03	1.03	186	1.33	1.04
		140–159	217	1.17	1.04	158	1.37	1.04
		≥160	36	1.17	1.12	35	1.61	1.11
Hypertension treatment								
		Yes	281	1.00	1.04	262	1.37	1.03
		No	2070	0.90	1.02	2075	1.03	1.02
Total cholesterol (mg/dL)								
		<160	481	0.88	1.03	486	0.99	1.03
		160–199	1027	0.90	1.02	1055	1.05	1.02
		200–239	642	0.94	1.03	602	1.11	1.02
		240–279	174	0.96	1.05	158	1.22	1.05
		≥280	27	1.01	1.17	36	1.20	1.18
	p for linear trend			0.023			<0.001	
HDL cholesterol (mg/dL)								
		≥60	398	0.98	1.04	836	0.99	1.02
		50–59	586	0.85	1.03	708	1.08	1.03
		40–49	870	0.89	1.02	603	1.12	1.03
		<40	497	0.96	1.03	190	1.17	1.04
Cotinine-verified current smoker								
		Ucot <50 (ng/mL)	1157	0.70	1.02	2053	1.04	1.02
		Ucot ≥50 (ng/mL)	1194	1.16	1.02	284	1.25	1.04
Total			2351	0.91	1.02	2337	1.06	1.01

All values were accounted for in study weights.

GM: geometric mean; GSE: geometric standard error.

Table 2 shows the Framingham estimate of 10 year CHD risk by age and blood cadmium quartiles and their linear trends. The means of the Framingham estimate of 10 year CHD risk were higher among Korean men than Korean women. For men, the means of the Framingham estimate of 10 year CHD risk increased with increasing blood cadmium quartiles (*p* for linear trend <0.05). For women, significantly increasing trends were observed in groups aged 20 to <35, 45 to <50, and 60 to <65 years.

**Table 2:** The means and standard errors of the Framingham estimate of 10-year CHD risk by age and heavy metal quartile.

Cadmium levels		Men				Women			
		n	Mean	SE	p for linear trend	n	Mean	SE	p for linear trend
20≤Age<35									
	1Q	148	1.09	0.04	<0.001	153	0.01	0.01	0.033
	2Q	148	1.26	0.07		153	0.02	0.01	
	3Q	150	1.66	0.13		154	0.04	0.02	
	4Q	149	1.43	0.07		155	0.08	0.03	
35≤Age<40									
	1Q	85	2.79	0.40	<0.001	82	0.20	0.10	0.458
	2Q	90	2.63	0.35		82	0.05	0.03	
	3Q	87	4.56	0.43		82	0.10	0.03	
	4Q	88	5.54	0.63		82	0.32	0.09	
40 ≤ Age<45									
	1Q	79	2.41	0.25	<0.001	71	0.29	0.14	0.929
	2Q	79	4.02	0.40		72	0.11	0.05	
	3Q	79	5.39	0.51		71	0.20	0.06	
	4Q	79	6.42	0.35		72	0.25	0.06	
45≤Age<50									
	1Q	70	4.82	0.58	<0.001	78	0.28	0.09	0.010
	2Q	70	4.71	0.45		78	0.42	0.08	
	3Q	71	6.51	0.76		78	0.67	0.14	
	4Q	71	10.02	1.13		78	1.16	0.36	
50≤Age<55									
	1Q	76	6.97	0.51	<0.001	78	1.07	0.11	0.456
	2Q	76	7.66	0.48		79	0.96	0.11	
	3Q	77	8.50	0.57		79	1.25	0.11	
	4Q	77	9.46	0.47		79	1.09	0.11	
55≤Age<60									
	1Q	70	8.08	0.64	0.002	71	1.84	0.23	0.293
	2Q	72	9.60	0.78		71	1.78	0.28	
	3Q	71	11.81	1.20		71	1.68	0.21	
	4Q	72	12.93	1.54		72	2.36	0.38	
60≤Age<65									
	1Q	54	11.38	0.57	<0.001	49	2.61	0.23	0.033
	2Q	54	11.18	0.51		49	2.24	0.20	
	3Q	54	12.99	0.57		49	3.21	0.24	
	4Q	55	14.52	0.70		49	3.74	0.54	

All values were accounted for in study weights.

The relationships between blood cadmium levels and the Framingham estimate of 10 year CHD risk by age groups among Korean men and women are shown in Table 3. For Korean men, the Framingham estimate of 10 year CHD risk was positively related to log-transformed blood cadmium levels (*p*<0.05) in all age groups. For Korean women, the Framingham estimate of 10 year CHD risk was positively related to log-transformed blood cadmium levels in those aged 45 to <50 years (*p*<0.05).

**Table 3:** Regression coefficients of log-transformed blood cadmium levels with the Framingham estimate of 10-year CHD risk among Korean.

Dependent variables	Men			Women		
	beta	SE	p value	beta	SE	p value
20≤Age<35	0.254	0.056	<0.001	0.039	0.020	0.057
35≤Age<40	1.731	0.416	<0.001	0.212	0.181	0.244
40≤Age<45	2.861	0.318	<0.001	−0.016	0.098	0.872
45≤Age<50	3.229	0.825	<0.001	0.549	0.213	0.011
50≤Age<55	1.979	0.378	<0.001	0.151	0.127	0.234
55≤Age<60	3.534	1.091	0.001	0.229	0.365	0.531
60≤Age<65	2.471	0.576	<0.001	0.891	0.571	0.120

All regression analyses were adjusted for survey year.

Results were estimated with study weights.

Table 4 shows the relationships between blood cadmium levels and each component of the Framingham estimate of 10 year CHD risk by age groups, after adjusting for survey years, age, and urinary cotinine. The log-transformed blood cadmium levels among men aged 20 to <35, 40 to <45, 50 to <55, and 60 to <65 years were found to positively correlate with systolic blood pressure (*p*<0.05), but not with total cholesterol or HDL cholesterol concentration. Log-transformed blood cadmium concentrations also correlated with urinary cotinine concentrations.

**Table 4:** Regression coefficients of log-transformed blood cadmium levels with each components the Framingham estimate of 10-year CHD risk among Korean men.

Dependent variables		beta	SE	p value	Dependent variables		beta	SE	p value
**20≤Age<35**					**50≤Age<55**				
	Age	0.702	0.215	0.001		Age	0.242	0.152	0.114
	SBP*	2.539	1.276	0.047		SBP*	7.999	1.958	<0.001
	Total cholesterol*	0.889	2.615	0.734		Total cholesterol*	−3.593	4.964	0.470
	HDL cholesterol*	1.258	1.027	0.221		HDL cholesterol*	1.216	1.316	0.356
	Urinary cotinine	832.616	80.250	<0.001		Urinary cotinine	1010.182	128.276	<0.001
**35≤Age<40**					**55≤Age<60**				
	Age	−0.069	0.148	0.643		Age	−0.013	0.146	0.932
	SBP*	1.972	1.553	0.205		SBP*	3.650	1.940	0.061
	Total cholesterol*	−2.082	3.439	0.545		Total cholesterol*	2.091	5.408	0.699
	HDL cholesterol*	0.145	1.334	0.914		HDL cholesterol*	1.765	1.443	0.223
	Urinary cotinine	732.704	77.385	<0.001		Urinary cotinine	809.187	112.660	<0.001
**40≤Age<45**					**60≤Age<65**				
	Age	−0.113	0.180	0.528		Age	0.189	0.214	0.378
	SBP*	7.085	3.211	0.028		SBP*	7.218	2.464	0.004
	Total cholesterol*	6.893	6.363	0.280		Total cholesterol*	8.403	5.785	0.148
	HDL cholesterol*	2.714	1.870	0.148		HDL cholesterol*	1.897	1.687	0.262
	Urinary cotinine	1012.800	117.604	<0.001		Urinary cotinine	790.009	126.085	<0.001
**45≤Age<50**									
	Age	0.384	0.145	0.009					
	SBP*	3.893	2.308	0.093					
	Total cholesterol*	4.484	4.897	0.361					
	HDL cholesterol*	−0.499	1.466	0.734					
	Urinary cotinine	971.939	126.092	<0.001					

Results were estimated with study weights.

beta: regression coefficients; SE: standard error; SBP: systolic blood pressure; HDL: high density lipoprotein.

Units: SBP (mmHg); Age (year); Total cholesterol (mg/dL); HDL cholesterol (mg/dL); Urinary cotinine (ng/mL).

All regression analyses were adjusted for survey year in multivariate regression analysis.

*: adjusted for survey years, age and urinary cotinine in multivariate regression analysis.

The relationship between blood cadmium levels and each component of the Framingham estimate of 10 year CHD risk by age groups among Korean women are shown in Table 5. After adjusting for survey years, age, and urinary cotinine, the log-transformed blood cadmium levels among women aged 20 to <35, 45 to <50, and 50 to <55 years were found to positively correlate with systolic blood pressure (*p*<0.05), but not with total or HDL cholesterol concentrations. Urinary cotinine concentration also correlated with log-transformed blood cadmium levels in women aged 20 to <35, 35 to <40, 45 to <50, and 60 to <65 years.

**Table 5:** Regression coefficients of log-transformed blood cadmium levels with each components the Framingham estimate of 10-year CHD risk among Korean women.

Dependent variables		beta	SE	p value	Dependent variables		beta	SE	p value
**20≤Age<35**					**50≤Age<55**				
	Age	1.086	0.246	<0.001		Age	−0.169	0.165	0.306
	SBP*	2.006	0.861	0.017		SBP*	5.872	2.429	0.016
	Total cholesterol*	−3.665	2.585	0.157		Total cholesterol*	−3.057	5.134	0.552
	HDL cholesterol*	−0.848	0.980	0.387		HDL cholesterol*	−0.587	1.486	0.693
	Urinary cotinine	173.655	36.654	<0.001		Urinary cotinine	44.197	23.852	0.065
**35≤Age<40**					**55≤Age<60**				
	Age	0.029	0.157	0.856		Age	0.106	0.209	0.613
	SBP*	2.602	1.531	0.090		SBP*	3.723	2.302	0.107
	Total cholesterol*	−2.216	7.422	0.766		Total cholesterol*	3.563	5.349	0.506
	HDL cholesterol*	0.959	1.716	0.577		HDL cholesterol*	−2.636	1.524	0.085
	Urinary cotinine	180.985	54.994	<0.001		Urinary cotinine	−0.518	59.251	0.993
**40≤Age<45**					**60≤Age<65**				
	Age	0.499	0.182	0.007		Age	−0.160	0.244	0.515
	SBP*	−0.097	1.998	0.961		SBP*	−0.818	3.235	0.824
	Total cholesterol*	−4.786	4.759	0.316		Total cholesterol*	12.705	6.998	0.071
	HDL cholesterol*	−0.099	1.751	0.955		HDL cholesterol*	−1.923	2.104	0.362
	Urinary cotinine	67.187	53.518	0.211		Urinary cotinine	182.213	86.820	0.037
**45≤Age<50**									
	Age	−0.045	0.197	0.819					
	SBP*	5.841	2.289	0.011					
	Total cholesterol*	−3.726	4.741	0.433					
	HDL cholesterol*	−0.150	1.809	0.934					
	Urinary cotinine	213.596	69.155	0.002					

Results were estimated with study weights.

beta: regression coefficients; SE: standard error; SBP: systolic blood pressure; HDL: high density lipoprotein.

Units: SBP (mmHg); Age (year); Total cholesterol (mg/dL); HDL cholesterol (mg/dL); Urinary cotinine (ng/mL).

All regression analyses were adjusted for survey year in multivariate regression analysis.

*: adjusted for survey years, age and urinary cotinine in multivariate regression analysis.

## Discussion

This study found that the Framingham estimate of 10 year CHD risk was positively associated with log-transformed blood cadmium concentrations in Korean men. Furthermore, log-transformed blood cadmium in specific age groups was positively correlated with systolic blood pressure, after adjusting for survey year, age, and urinary cotinine concentration.

Age was found to be the most powerful factor influencing the Framingham estimated 10 year CHD risk, with Framingham point scores ranging from −9 in subjects aged 20–34 years to 13 in subjects aged 75–79 years [11]. A study of subjects aged ≥70 years, thus controlling for the effect of age on the Framingham estimate of 10 year CHD risk, did not show associations in all age groups [10]. In the present study, the association between the Framingham estimate of 10 year CHD risk and blood cadmium concentration was analyzed in subjects aged ≤65 years. To avoid bias due to the wide range in ages, subjects were stratified into seven age groups (20–34, 35–39, 40–44, 45–49, 50–54, 55–59, and 60–64 years), and the Framingham estimated 10 year CHD risk was assessed in each.

Smoking may be an important confounder of both blood cadmium concentrations and Framingham estimated 10 year CHD risk. To elucidate the association between these two parameters, without considering the effect of smoking, an additional sensitivity test was performed (Table S1). Although a positive association was observed between blood cadmium concentrations and Framingham estimated 10 year CHD risk, the association was not statistically significant, suggesting that smoking may be a confounder. However, cotinine-verified non-smokers frequently include former smokers. An additional analysis was performed for questionnaire-verified current smokers vs. never smokers (Table S2), with the results suggesting a positive association between log-transformed blood cadmium levels and Framingham estimated 10 year CHD risk score in questionnaire-verified never smokers among Korean males aged 40 to <45 and 55 to <60 years (*p*<0.01). Thus, the blood cadmium levels per se may be related to risks of CHD. To assess the role of smoking in this patient cohort, the association between blood cadmium and Framingham estimated 10 year CHD risk was analyzed, after stratification for smoking status (Table S3). The regression coefficients for log-transformed blood cadmium levels with the Framingham estimate of 10 year CHD risk among Korean male non-smokers and smokers were 1.739 (*p*<0.001) and 3.172 (*p*<0.001), respectively. The correlations between blood cadmium and Framingham 10 year CHD risk among non-smokers suggest that smoking may play a modifying rather than a confounding role in this patient population. The results in Tables S1 and S2, however, should be interpreted cautiously, due to the small numbers of never smokers and a possible overestimation of the effects of smoking due to information bias. Previous studies of hidden smoking among Koreans [14] and overestimation of the health effect by questionnaire-verified smoking status [16] suggested caution in interpreting health effects related to questionnaire-verified smoking status. Further studies are needed to overcome these limitations.

Urinary cotinine has been found to correlate positively with blood cadmium levels [17]. A recent study found that self-reported smoking rates in Korean men and women were 5.3% and 8.0%, respectively, lower than cotinine-verified smoking rates [14]. The reported sensitivity for self-reported smoking status was 87.9% for men and 41.1% for women [14]. Thus, urinary cotinine concentrations were measured to verify current smokers, thus avoiding information bias. In evaluating the association between the components of the Framingham point scores and blood cadmium, we adjusted for urinary cotinine levels in regression analysis.

There is evidence for the biological effects of cadmium as influencing the Framingham estimate of 10 year CHD risk. Our study found that non-occupational cadmium exposure may be associated with the Framingham estimate of 10 year risk by elevating systolic blood pressure. Studies have assessed the association between non-occupational cadmium exposure and blood pressure [18]–[23]. Cadmium has been found to increase blood pressure through several mechanisms, including acting as a partial agonist for calcium channels, inhibiting vasodilation [22], having a direct vasoconstrictor action via oxidative stress [21], depleting glutathione, and altering sulfhydryl homeostasis in the vascular wall [19], [20], [23]. Epidemiologic studies have demonstrated that chronic exposure to cadmium may be related to blood pressure in the general population [7], [24]. In a previous study of the general Korean population, the odds ratio for hypertension in the highest vs. the lowest quartile of blood cadmium level was 1.52 (95% CI: 1.13–2.05) [7]. An association between blood cadmium levels and a modest elevation in blood pressure was also shown in the general US population [24]. However, in that study, hypertension was defined as a binomial variable (presence or absence). Binomial variable analysis, however, may result in information bias, since it depends on an interviewee's memory or intention. To overcome any possible misclassification bias (presence or absence of hypertension), we assessed blood pressure as a continuous variable in multiple survey regression analyses, finding significant positive relationships between log-transformed cadmium levels in blood and systolic blood pressure among several age groups, after adjusting for survey year, age, and smoking.

We observed gender differences in the association between the Framingham estimate of 10 year CHD risk and blood cadmium levels. The Framingham estimate of 10 year CHD risk was lower in women, and the range of estimates in women was narrower than in men. For women aged 45–49 years, however, the Framingham estimate of 10 year CHD risk and systolic blood pressure were associated with blood cadmium levels, suggesting that hormonal changes may make the influence of cadmium more prominent in perimenopausal women. Korean women aged ≥45 years had higher (≥1.30 µg/L) blood cadmium levels than those aged <45 years. Interestingly, the Framingham estimate of 10 year CHD risk for women was likely to increase at a similar age. Metallothionein (MT) plays a protective role in cadmium toxicity [17]. Animal studies have shown that female hormones, such as 17 beta-estradiol and progesterone, could reduce the inhibition of MT mRNA expression [25]. Therefore, changes in female hormones may reduce the protective effect of MT on cadmium exposure during perimenopause. Moreover, diseases that influence the Framingham estimate of 10 year CHD risk, such as elevated blood pressure, may occur at a certain cadmium threshold level, but additional studies are required.

Another possible cause of gender difference influencing the Framingham estimate of 10 year CHD risk is smoking. In a study examining the relationship between the Framingham estimate of 10 year CHD risk and non-occupational heavy metal levels among elderly Swedish subjects, blood cadmium was related to Framingham risk score among women, but not men [10], a finding inconsistent with our study results. The proportion of current smokers was higher in Swedish females than in males (11.4% vs. 9.9%) [10], whereas the proportion of cotinine-verified smokers was much higher in Korean males than in females (52.2% vs. 12.1%). Smoking is related to blood cadmium levels as well as to the Framingham estimate of 10 year CHD risk and its components [3], [7], [24], [26]. Therefore, differences in current smoking status may have influenced the primary results of both the previous study [10] and ours. A previous study concerning hidden female smoking found that the sensitivity of the interview was <50% among Korean females [14]. To overcome this information bias, urinary cotinine concentration was used to verify smoking status, with urinary cotinine and blood cadmium levels being linearly associated in each age group of females in the present study.

Our study has several limitations due to its cross-sectional study design. First, subjects in our study were recruited in three different years. Despite efforts to ensure a representative population in each year, there may have been possible selection bias. To reinforce the power of the estimation of results, we used KNHANES from three different years. The Korea Center for Disease Control and Prevention has provided a statistical method of evaluating the results of 3 years in a dataset, using an adjusting equation for sampling weights [12]. We utilized these methods to generate new weights and estimated the regression coefficients in multiple survey regression analysis. In addition, we also adjusted for survey year. Second, this national survey did not make use of laboratory tests for chronic heavy metal exposure such as urinary cadmium. This limitation should be considered in interpreting our study results. Although blood cadmium levels do not reflect lifetime exposure, the markers used were not only the most valid biomarkers for recent cadmium exposure but also reflect the slow components of heavy metal exposure, since cadmium and lead are released into the blood from bone or other organs [27], [28]. However, this inevitable limitation should be considered carefully. Third, the Framingham estimate of 10 year CHD risk was derived from a white US population rather than from Koreans. Therefore, further evaluation should utilize a CHD risk scale derived from the Korean population.

Despite these limitations, our study has several strengths. First, as age is the most powerful influencing factor in the Framingham estimate of 10 year CHD risk, we controlled for age by stratifying subjects into seven age groups for multivariate analysis. Thus, the present study was able to control for the potential confounding effect of age. Second, the large number of subjects in our study may provide a higher statistical power than that of a previous study of elderly Swedish subjects [10]. Third, since there may be a high false negative rate with regard to current smoking as assessed by questionnaire, we verified current smoking status and quantified the degree of smoking using a biologic marker (urinary cotinine) [14]. Fourth, we observed a relationship between blood cadmium and systolic blood pressure. Previous laboratory studies have demonstrated the biologic plausibility of this association [20], [23]. Therefore, low-level cadmium exposure may influence the Framingham estimate of 10 year CHD risk by elevating blood pressure. Furthermore, our study subjects were from a healthy and community-based sample representative of the general Korean population, allowing our results to be generalized to the Korean population.

## Conclusions

Low level cadmium exposure may be associated with CHD risk in men. Overall non-occupational cadmium exposure has been declining for several decades [29], Efforts to reduce non-occupational cadmium exposure, by reinforcing tobacco control and reducing the use of metal-containing fertilizers, may reduce metal-related CHD in the general population.

## Supporting Information

Table S1 **Additional analysis for regression coefficients of log-transformed blood cadmium levels with the Framingham estimate of 10-year CHD risk from** **table 3** **by cotinine verified smoking status.**
(DOCX)

Table S2 **Additional analysis for regression coefficients of log-transformed blood cadmium levels with the Framingham estimate of 10-year CHD risk from** **table 3** **by questionnaire verified smoking status (current smoker and never-smoker).**
(DOCX)

Table S3 **Regression coefficients of log-transformed blood cadmium levels with the Framingham estimate of 10-year CHD risk by smoking status.**
(DOCX)
