# Supplementary material for: Association between Blood Cadmium Levels and 10-Year Coronary Heart Disease Risk in the General Korean Population: The Korean National Health and Nutrition Examination Survey 2008–2010
Source: PLoS One. 2014 Nov 10;9(11):e111909. doi: 10.1371/journal.pone.0111909 (PMC4226505; doi:10.1371/journal.pone.0111909)
Supplement: Table S1 — Additional analysis for regression coefficients of log-transformed blood cadmium levels with the Framingham estimate of 10-year CHD risk from table 3 by cotinine verified smoking status. (DOCX) [file pone.0111909.s001.docx]

Supporting information

Table S1. Additional analysis for regression coefficients of log-transformed blood cadmium levels with the Framingham estimate of 10-year CHD risk from table 3 by cotinine verified smoking status

| Dependent variables | Smokers  (urinary cotinine ≥ 50 ng/mL) | | | | |  | Non-smokers  (urinary cotinine < 50 ng/mL) | | | | |
| --- | --- | --- | --- | --- | --- | --- | --- | --- | --- | --- | --- |
|  | Men | |  | Women | |  | Men | |  | Women | |
|  | Beta | p value |  | beta | p value |  | beta | p value |  | beta | p value |
| 20≤ Age <35 | 0.034 | <0.001 |  | 0.023 | 0.813 |  | 0 | . |  | 1.48$\times$E^-53^ | <0.001 |
| 35≤ Age <40 | -0.760 | 0.365 |  | 0.773 | 0.270 |  | -0.110 | 0.214 |  | -0.105 | 0.095 |
| 40≤ Age <45 | -0.115 | 0.854 |  | -0.905 | 0.170 |  | 0.395 | 0.169 |  | 0.019 | 0.321 |
| 45≤ Age <50 | 0.555 | 0.627 |  | 2.306 | 0.068 |  | 0.110 | 0.830 |  | 0.016 | 0.802 |
| 50≤ Age <55 | -1.105 | 0.169 |  | 0.065 | 0.605 |  | 0.309 | 0.282 |  | 0.151 | 0.234 |
| 55≤ Age <60 | 4.018 | 0.068 |  | 1.640 | 0.245 |  | -0.665 | 0.397 |  | -0.009 | 0.980 |
| 60≤ Age <65 | 1.573 | 0.147 |  | 1.758 | 0.283 |  | 0.327 | 0.567 |  | 0.580 | 0.333 |
| All regression analyses were adjusted for survey year  Results were estimated with study weights | | | | | | | | | | | |
