# Supplementary material for: Association between Blood Cadmium Levels and 10-Year Coronary Heart Disease Risk in the General Korean Population: The Korean National Health and Nutrition Examination Survey 2008–2010
Source: PLoS One. 2014 Nov 10;9(11):e111909. doi: 10.1371/journal.pone.0111909 (PMC4226505; doi:10.1371/journal.pone.0111909)
Supplement: Table S2 — Additional analysis for regression coefficients of log-transformed blood cadmium levels with the Framingham estimate of 10-year CHD risk from table 3 by questionnaire verified smoking status (current smoker and never-smoker). (DOCX) [file pone.0111909.s002.docx]

Supporting information

Table S2. Additional analysis for regression coefficients of log-transformed blood cadmium levels with the Framingham estimate of 10-year CHD risk from table 3 by questionnaire verified smoking status (current smoker and never-smoker)

| Dependent variables | Current smokers | | | | |  | never-smokers | | | | |
| --- | --- | --- | --- | --- | --- | --- | --- | --- | --- | --- | --- |
|  | Men | |  | Women | |  | Men | |  | Women | |
|  | Beta | p value |  | beta | p value |  | beta | p value |  | beta | p value |
| 20≤ Age <35 | 0.007 | 0.942 |  | 0.153 | 0.268 |  | 0.027 | 0.094 |  | 0.015 | 0.392 |
| 35≤ Age <40 | 0.007 | 0.993 |  | 0.521 | 0.250 |  | 0.350 | 0.354 |  | 0.098 | 0.606 |
| 40≤ Age <45 | 0.297 | 0.662 |  | -1.391 | 0.059 |  | 1.861 | 0.007 |  | 0.035 | 0.460 |
| 45≤ Age <50 | 0.412 | 0.807 |  | 4.782 | 0.038 |  | -0.037 | 0.972 |  | 0.104 | 0.336 |
| 50≤ Age <55 | -1.331 | 0.144 |  | 1.134 | 0.001 |  | 0.754 | 0.077 |  | 0.046 | 0.717 |
| 55≤ Age <60 | 4.266 | 0.086 |  | -1.433 | 0.466 |  | 1.695 | 0.003 |  | 0.045 | 0.895 |
| 60≤ Age <65 | 1.246 | 0.335 |  | 2.607 | 0.121 |  | 1.085 | 0.497 |  | 0.523 | 0.387 |
| All regression analyses were adjusted for survey year  Results were estimated with study weights | | | | | | | | | | | |
