# Supplementary material for: Association between Blood Cadmium Levels and 10-Year Coronary Heart Disease Risk in the General Korean Population: The Korean National Health and Nutrition Examination Survey 2008–2010
Source: PLoS One. 2014 Nov 10;9(11):e111909. doi: 10.1371/journal.pone.0111909 (PMC4226505; doi:10.1371/journal.pone.0111909)
Supplement: Table S3 — Regression coefficients of log-transformed blood cadmium levels with the Framingham estimate of 10-year CHD risk by smoking status. (DOCX) [file pone.0111909.s003.docx]

Supporting information

**Table S3.** Regression coefficients of log-transformed blood cadmium levels with the Framingham estimate of 10-year CHD risk by smoking status

|  | Men | |  | Women | |
| --- | --- | --- | --- | --- | --- |
|  | Beta | p value |  | Beta | p value |
| Smoker | 3.172 | <0.001 |  | 1.391 | <0.001 |
| Non-smoker | 1.740 | <0.001 |  | 0.456 | <0.001 |
| Overall | 3.203 | <0.001 |  | 0.613 | <0.001 |
